# Interspecific variation and elevated CO_2_ influence the relationship between plant chemical resistance and regrowth tolerance

**DOI:** 10.1002/ece3.6284

**Published:** 2020-05-17

**Authors:** Leslie E. Decker, Mark D. Hunter

**Affiliations:** ^1^ Department of Biology Stanford University Stanford CA USA; ^2^ Department of Ecology and Evolutionary Biology University of Michigan Biological Sciences Building Ann Arbor MI USA

**Keywords:** *Asclepias*, cardenolides, global change ecology, plant‐herbivore interactions, resistance to herbivory

## Abstract

To understand how comprehensive plant defense phenotypes will respond to global change, we investigated the legacy effects of elevated CO_2_ on the relationships between chemical resistance (constitutive and induced via mechanical damage) and regrowth tolerance in four milkweed species (*Asclepias*). We quantified potential resistance and tolerance trade‐offs at the physiological level following simulated mowing, which are relevant to milkweed ecology and conservation. We examined the legacy effects of elevated CO_2_ on four hypothesized trade‐offs between the following: (a) plant growth rate and constitutive chemical resistance (foliar cardenolide concentrations), (b) plant growth rate and mechanically induced chemical resistance, (c) constitutive resistance and regrowth tolerance, and (d) regrowth tolerance and mechanically induced resistance. We observed support for one trade‐off between plant regrowth tolerance and mechanically induced resistance traits that was, surprisingly, independent of CO_2_ exposure. Across milkweed species, mechanically induced resistance increased by 28% in those plants previously exposed to elevated CO_2._ In contrast, constitutive resistance and the diversity of mechanically induced chemical resistance traits declined in response to elevated CO_2_ in two out of four milkweed species. Finally, previous exposure to elevated CO_2_ uncoupled the positive relationship between plant growth rate and regrowth tolerance following damage. Our data highlight the complex and dynamic nature of plant defense phenotypes under environmental change and question the generality of physiologically based defense trade‐offs.

## Graphical abstract

To understand how comprehensive plant defense phenotypes will respond to global change, we investigated the legacy effects of elevated CO_2_ on the relationships between chemical resistance and regrowth tolerance in four milkweed species (*Asclepias*). We found interspecific variation among milkweed species influenced the relationship between mechanically induced chemical resistance and regrowth tolerance. Previous exposure to elevated CO_2_ increased mechanically induced resistance by 28% and uncoupled the positive relationship between plant growth rate and regrowth tolerance following damage.

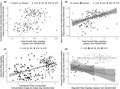

## 1 INTRODUCTION

Plants employ a suite of defensive traits to deter and minimize the impacts of herbivory (Agrawal & Fishbein, 2006). As a result, critical factors that contribute to plant fitness in the context of damage may be overlooked when defenses are studied in isolation (Baucom & De Roode, 2011). Regrowth tolerance and chemical resistance are two major strategies of defense that plants employ against herbivory and that jointly influence fitness (Agrawal, 2011;Fornoni, 2011;Núñez‐Farfán, Fornoni, & Valverde, 2007;Stamp, 2003;Strauss & Agrawal, 1999;Zas, Moreira, & Sampedro, 2011). One major form of defense, resistance to herbivory, occurs through physical and chemical traits such as trichomes, latex exudation, thorns, and toxic secondary metabolites that together reduce herbivore performance (Rhoades, 1985). Multiple resistance traits can be both constitutively expressed before damage and induced following damage; however, chemical resistance is perhaps best known for this temporal strategy (Agrawal & Karban, 1999;Karban & Baldwin, 2013). Because resistance traits are themselves metabolically costly (Bekaert, Edger, Hudson, Pires, & Conant, 2012;Gershenzon, 1994;Strauss, Rudgers, Lau, & Irwin, 2002), it is thought that these costs manifest in the form of trade‐offs with other plant functions like growth rates (Agrawal, 2011;Fineblum, Rausher, & D., 1995;van der Meijden, Wijn, & Verkaar, 1988;Stamp, 2003). Plant tolerance to herbivory, or compensatory growth following damage, minimizes fitness losses through simultaneous shifts in physiology and resource allocation (Fornoni, Ez‐Farfán, & J, Valverde PL, 2003;Rosenthal & Kotanen, 1994;Strauss & Agrawal, 1999).

Numerous hypotheses have been developed to predict resource allocation to the competing plant functions of chemical resistance and regrowth tolerance, often with variable generality and empirical support (*reviewed in* Stamp, 2003). On a macroevolutionary level, the resource availability hypothesis (RAH) predicts that high‐resource environments select for fast‐growing species that invest in regrowth tolerance following damage rather than chemical defense production (Coley & Chapin, 1985;Endara & Coley, 2011). But fewer hypotheses address the interplay of tolerance and chemical defense within populations (Hahn, Agrawal, Sussman, & Maron, 2019;Hahn & Maron, 2016) or physiologically within the lifetime of individuals. At the cellular and tissue level, the growth–differentiation balance hypothesis (GDB) posits that plants in high‐resource environments will not be limited by photosynthesis and will allocate more energy into regrowth rather than into cellular differentiation‐related processes such as secondary metabolism (Herms & Mattson, 1992). At intermediate resource conditions, the GDB predicts that plants will be limited in growth but not photosynthetic capacity and will produce more secondary metabolites relatively cheaply. Due to the need to test at multiple resource levels, and measure not only growth rate, but net assimilation and secondary metabolism, the GDB has proven difficult to test but still provides a useful framework of plant defense at the physiological level (Stamp, 2004). In general, trade‐offs between tolerance and chemical resistance arise as a result of plant allocation strategies meant to optimize fitness in a variable environment (Züst & Agrawal, 2017). Therefore, understanding the environmental conditions under which trade‐offs manifest is of critical importance.

The rapidly rising concentration of atmospheric carbon dioxide is well‐known to influence chemical resistance to herbivores and plant growth rates. Both the composition and concentration of constitutively expressed and induced plant secondary metabolites change in response to elevated CO_2_ depending on the class of compounds considered (Bidart‐Bouzat, Mithen, & Berenbaum, 2005;Hunter, 2001;Jia, Zhao, Liu, & Huang, 2016;Klaiber, Dorn, & Najar‐Rodriguez, 2013;Robinson, Ryan, & Newman, 2012;Ryan, Rasmussen, & Newman, 2010;Zavala, Nabity, & DeLucia, 2013). Further, elevated CO_2_ suppresses the synthesis of jasmonic acid and stimulates the production of salicylic acid, compromising the plant's ability to mount an induced resistance response (Ode, Johnson, & Moore, 2014).

Changes in phytohormonal signaling pathways also mediate plant growth and regrowth tolerance following damage under elevated CO_2_ (Guo et al., 2012). In general, by increasing photosynthesis and water use efficiency, elevated CO_2_ positively affects plant growth rates (Drake, Gonzalez‐Meler, & Long, 1997; Ainsworth & Long, 2005; Robinson et al., 2012; Bazzaz, Ackerly, Woodward, & Rochefort, 2013). However, the direct effects of elevated CO_2_ on plant regrowth tolerance following damage can be negative (Guo et al., 2012;Lau & Tiffin, 2009;Marshall, Avila‐Sakar, & Reekie, 2008;Wilsey, 2001) partially because of increased nutrient limitation under elevated CO_2_ paired with phytohormonal suppression. Studies that explore the integrated influence of elevated CO_2_ on the relationships between resistance and tolerance are sorely lacking.

Even less is known about the lingering effects of past CO_2_ enrichment on plants. Though not ecologically plausible, the modulation of exposure to environmental change drivers such as elevated CO_2_ partially reveals energetic allocation decisions made by plants under future conditions, and the persistence of those responses. Extrapolations based on the substantial below‐ground carbon sink and increased soil microbial turnover that develops in response to elevated CO_2_ predict mixed but lingering effects of elevated CO_2_ on plant regrowth tolerance (Hungate, Johnson, & Dijkstra, 2006; Stiling, Moon, & Rossi, 2013). To our knowledge, only two studies have examined plant responses to elevated CO_2_ beyond the cessation of enrichment and found lasting effects on aspects of root morphology such as fine root hairs (Stiling et al., 2013) and increases in regrowth tolerance following fire (Bain & Day, 2019). These studies follow plant and arthropod communities in the years following enrichment cessation, yet how plant physiological properties will respond to abrupt changes in CO_2_ enrichment over the course of a growing season remains to be tested.

Here, we investigate the legacy effects of elevated CO_2_ on the chemical resistance traits and regrowth tolerance of four milkweed species (*Asclepias*). Specifically, we examined the effects of elevated CO_2_ on four hypothesized trade‐offs between the following: (a) initial growth rate and constitutive chemical resistance, (b) initial growth rate and mechanically induced chemical resistance, (c) constitutive chemical resistance and regrowth tolerance following damage, and (d) regrowth tolerance and mechanically induced chemical resistance. To our knowledge, no theory exists to predict the interaction between resistance and regrowth tolerance strategies under changing carbon supplementation. Nevertheless, we predicted that elevated CO_2_ would induce higher growth rates and regrowth rates and depress constitutive secondary metabolites following the GDB hypothesis and mitigate, in part, any trade‐off between chemical resistance traits and regrowth tolerance in milkweed. By analyzing changes in plant tolerance and resistance chemistry, we aimed to improve our understanding of how future environmental conditions may influence the defensive phenotype of plants, with implications for the herbivore communities that damage them.

## 2 MATERIALS AND METHODS

### 2.1 Study system

The four milkweed, *Asclepias,* species used in our study (*A. syriaca, A. speciosa, A. incarnata,* and *A. curassavica*) originate from North and Central America (Woodson, 1954) and support herbivores that range from phloem‐feeding insects such as oleander aphids (*Aphis nerii)* to chewing insects capable of removing large amounts of tissue, like monarch caterpillars (*Danaus plexippus*), and long horn beetles (*Tetraopes* spp.). Most milkweed herbivores specialize within the genus because *Asclepias* produce a well‐characterized suite of defenses against herbivory.

To physically deter feeding by arthropod herbivores, milkweed plants exude latex, produce trichomes, and increase leaf toughness (Agrawal & Fishbein, 2006;Agrawal & Konno, 2009;Hochwender, Marquis, & Stowe, 2000;Zalucki, Brower, & Alonso‐M, 2001). However, milkweeds are best known for synthesizing a class of toxic steroids known as cardenolides that disrupt Na^+^/K^+^‐ATPase in the Na^+^/K^+^‐channels of animal cells (Agrawal, Petschenka, Bingham, Weber, & Rasmann, 2012). The composition and concentration of cardenolides produced constitutively by milkweed plants vary substantially within and among milkweed species (Agrawal et al., 2012;Rasmann & Agrawal, 2011). Damage induces quick increases in cardenolide concentrations and changes in cardenolide composition (Malcolm & Zalucki, 1996). Regrowth following damage also plays a prominent role in the defensive phenotype of milkweeds (Agrawal & Fishbein, 2008;Tao, Ahmad, Roode, & Hunter, 2016). Despite a growing body of work illustrating the effects of environmental change on milkweed chemistry and milkweed growth (Matiella, 2012;Tao, Berns, & Hunter, 2014;Vannette & Hunter, 2011), no study to date has explored the interplay between milkweed chemical resistance traits (both constitutive and induced) and regrowth tolerance under future environmental conditions.

We grew four species of milkweed under ambient (400 ppm) and elevated (760 ppm) concentrations of atmospheric CO_2_ at the University of Michigan Biological Station (UMBS). To manipulate atmospheric CO_2_ concentrations, we used an outdoor array consisting of 40 open‐top chambers, with 20 chambers maintained at ambient CO_2_, and 20 chambers maintained at elevated CO_2_ from May through August of 2015. Chambers were 1 m high cubes with an octagonal top of diameter of 0.8 m composed of a PVC frame and clear plastic walls following a modified design of Drake, Leadley, Arp, Nassiry, and Curtis (1989).

We chose *Asclepias* species that vary in foliar cardenolide concentrations. Specifically, we included *A. incarnata* (low cardenolide), *A. speciosa, A. syriaca* (both medium cardenolide), and *A. curassavica* (high cardenolide). Seeds of *A. speciosa* and *A. curassavica* were obtained from commercial sources (Prairie Moon Nurseries, Winona, USA), and seeds of *A. incarnata* and *A. syriaca* were collected locally (Cheboygan county, MI). We surface‐sterilized all seeds following a six‐week cold stratification period (for all but tropical *A. curassavica*) and germinated seeds on moist filter paper for 1 week. We planted seedlings in 983 cm^3^ Deepots^TM^ (6.9 cm diameter by 35.6 cm height) containing Metromix 360 (Sun Gro Horticulture, Vancouver, BC) and Osmocote Controlled Release Fertilizer [N:P:K:16:16:16 ppm *N* (g/g)] (ICL Specialty Fertilizers, Dublin, USA) on May 5, 15. Germinated seedlings were watered daily and grown in the UMBS greenhouse for two weeks before they were moved to randomly assigned chambers in the CO_2_ array. Once in the array, potted plants were maintained under their CO_2_ treatments for three months. To minimize the entrance of herbivores into the chambers, we placed fine mesh coverings over the openings of each chamber and physically removed any herbivores that we observed during daily visual inspections.

Within each chamber, we grew as many as seven plants of each milkweed species. Low germination success limited the number of *A. speciosa* and *A. syriaca* used in this study, and not all milkweed species were represented in every chamber. Overall, our eight treatments (2 CO_2_ treatments × 4 milkweed species) combined for a total of 442 plants, with exact replicate numbers reported in Table 1.

**Table 1:** Sample sizes of 442 milkweed plants grown under either ambient (400 ppm) or elevated (760 ppm) CO_2_ grouped (a) by species and (b) by their distribution in 40 open‐top chambers. Species codes are as follows: CUR = A. curassavica, SYR = A. syriaca, SPE = A. speciosa, INC = A. incarnata

CO_2_ treatment	species	*N*
Ambient	*A. curassavica*	84
	*A. incarnata*	105
	*A. speciosa*	22
	*A. syriaca*	25
Elevated	*A. curassavica*	81
	*A. incarnata*	91
	*A. speciosa*	23
	*A. syriaca*	11

CO_2_ Treatment	Chamber	CUR	INC	SPE	SYR	Chamber	CUR	INC	SPE	SYR
Elevated	**1**	4	3	0	2	**21**	4	4	2	1
Ambient	**2**	6	6	1	2	**22**	5	6	2	0
Elevated	**3**	6	7	1	0	**23**	2	5	3	0
Ambient	**4**	3	6	2	3	**24**	5	5	0	1
Elevated	**5**	4	4	3	1	**25**	6	6	2	1
Ambient	**6**	4	4	0	1	**26**	4	6	1	0
Elevated	**7**	5	6	2	0	**27**	2	4	0	1
Ambient	**8**	4	6	1	1	**28**	4	6	0	1
Elevated	**9**	1	6	0	0	**29**	5	5	2	0
Ambient	**10**	4	5	3	1	**30**	3	5	0	2
Elevated	**11**	4	5	1	0	**31**	5	2	0	1
Ambient	**12**	4	6	2	1	**32**	4	3	0	0
Elevated	**13**	3	1	1	0	**33**	4	6	3	0
Ambient	**14**	2	5	2	3	**34**	4	5	0	1
Elevated	**15**	6	3	1	1	**35**	2	3	1	2
Ambient	**16**	5	5	0	3	**36**	6	6	3	2
Elevated	**17**	3	4	1	0	**37**	4	6	1	1
Ambient	**18**	5	4	0	0	**38**	5	6	2	1
Elevated	**19**	5	6	0	0	**39**	6	5	0	0
Ambient	**20**	5	6	2	1	**40**	2	4	0	1

Using a LI‐COR 320 IRGA (LI‐COR, Lincoln, USA), we monitored atmospheric CO_2_ concentrations daily in the 20 elevated CO_2_ chambers and in one randomly selected ambient CO_2_ chamber. Concentrations of CO_2_ were adjusted throughout the day to maintain the target of 760 ppm in each elevated chamber. The ambient temperature inside each chamber was recorded every hour using a thermochron datalogger (Thermochron, Baulkham Hills, Australia). Elevated CO_2_ chambers averaged 21.03 (±0.034) ºC, and ambient CO_2_ chambers averaged 21.24 (±0.038) ºC, roughly 2ºC higher than the outside average temperature of 18.93 (±0.039) ºC.

### 2.2 Simulated damage and growth measures

Three months following the initial transfer of plants into the array, we simulated clipping/mowing by cutting all plants at the soil line. Many milkweed habitats important to the specialist herbivores associated with milkweed are located near roadways and agricultural fields that are regularly mowed. Properly timed mowing can improve reproduction and decrease predator abundance of certain milkweed specialists, including the monarch butterfly (Haan & Landis, 2019). Thus, our simulated mowing represents an ecologically relevant stress regularly experienced by many milkweed plants. Moreover, at our field site in northern Michigan, we have observed chipmunks (*Tamius striatus*), milkweed stem weevils (*Rhyssomatus lineaticollis*), and porcupines (*Erethizon dorsatum*) all remove the entire above‐ground tissues of milkweed plants. Other herbivores such as monarch caterpillars, and milkweed tussock moths (*Euchaetes egle*), have also been observed to remove large amounts of foliage. Thus, our clipping treatment also represents severe but not infrequent levels of herbivore damage experienced by milkweed plants. We recognize that mechanical damage does not completely mimic actual herbivory because oral secretions and regurgitant released from the herbivore at the time of feeding can enter wounded plant tissue, inducing the release of jasmonic acid, a phytohormone critical to the production of defensive secondary metabolites (McCloud & Baldwin, 1997).

The aboveground biomass that we removed was dried at 60°C, weighed, and used to calculate growth rate prior to damage (below). Cut plants were watered, moved to the UMBS greenhouse, and maintained under identical (ambient CO_2_) conditions for three weeks due to external limitations on use of the chambers. However, by re‐growing clipped plants under ambient CO_2_ we are able to isolate the legacy effects of altered carbon availability prior to damage on regrowth tolerance, and potential trade‐offs between growth and resistance. Thus, we can examine the repercussions of previous energetic allocation decisions made by plants under carbon‐enriched conditions in comparison to those under ambient conditions. After a three‐week period, the aboveground regrowth plant material was harvested, dried at 60°C, and weighed as a measure of regrowth tolerance.

For a measure of growth rate prior to damage, we divided the aboveground dry biomass of the plant by 64 days (the number of days since the seedling had been transferred to soil) following Agrawal and Fishbein (2008). Similarly, to calculate plant regrowth rate following mechanical damage, we divided the mass of the regrowth material by 21 days (the length of time plants were allowed to regrow following damage). Differences in regrowth rate following damage are important for the competitive success and ultimate fitness of plants (Züst & Agrawal, 2017).

### 2.3 Chemical analyses and resistance classifications

We collected samples of the original aboveground foliage and the regrowth foliage of each plant for cardenolide analysis using established methods (Tao & Hunter, 2012;Vannette & Hunter, 2011;Zehnder & Hunter, 2009). Roughly, 20 mg of dried plant material was ground in a ball mill, deposited in 1 ml methanol, and stored at −10°C prior to analysis. Cardenolides were extracted, separated, and quantified with a 0.15mg/ml digitoxin internal standard (Sigma Chemical Company), by reverse‐phase high‐performance liquid chromatography (HPLC) on a Waters Acquity UPLC with PDA detector (Waters Corporation, Milford). Peaks with symmetrical absorbance between 217–222 nm were identified as cardenolides. Cardenolide concentrations were calculated as the sums of all separated peak areas, corrected by the concentration of the internal digitoxin standard and sample dry mass. We used digitoxin as an internal standard because it is absent from *Asclepias* and because purified standards remain unavailable for a majority of milkweed cardenolides. We recognize that cardenolides may differ in their concentration–area relationships, and our estimates of cardenolide concentration should be considered as measured in digitoxin‐equivalents. Because milkweed plants were grown in field mesocosms which excluded herbivores all season, the foliar cardenolides measured from plants prior to simulated damage represent natural levels of constitutive resistance. Conversely, the foliar cardenolide concentrations of regrown tissue following clipping represent mechanically induced resistance.

### 2.4 Statistical analyses

In all analyses that follow, we used either linear mixed models (LMMs; Lme4 package) or generalized linear mixed models (GLMMs; Lme4 package). To account for variation among chambers and the nonindependence of plants grown within the same individual chamber, we included chamber identity as a random effect in all of our models described below. This design allows us to test our hypotheses at the level of plant individuals to capture relevant variation in our analyses, while accounting for multiple plants within chambers. We performed all statistical tests in R version 3.6.0 (R Core Team, 2018) and selected models using likelihood ratio tests (Burnham & Anderson, 2002). Variables were transformed to best achieve normality of error as tested by the Shapiro–Wilk normality test. Homogeneity of variance and distribution of residuals were inspected using quantile–quantile and residuals fitted value plots to check for conformation to model assumptions (Crawley, 2012).

### 2.5 Testing for trade‐offs among milkweed growth, regrowth tolerance, and resistance chemistry

#### 2.5.1 Plant growth rate and chemical resistance before damage

We used an LMM with log‐transformed initial foliar cardenolide concentrations as the dependent variable and square root‐transformed growth rate prior to clipping, CO_2_ treatment, and milkweed species as fixed effects. An interaction between growth rate prior to clipping and CO_2_ indicates a difference between the CO_2_ treatments in the extent to which growth rate correlates with the production of cardenolides.

#### 2.5.2 Plant growth rate before damage and mechanically induced resistance of regrowth tissues

We used an LMM with log‐transformed foliar cardenolide concentrations of the regrowth foliage as the dependent variable and square root‐transformed growth rate prior to clipping, CO_2_ treatment, and milkweed species as fixed effects. An interaction between initial growth rate and CO_2_ indicates a difference between CO_2_ treatments in the potential trade‐off between plant growth rate before damage and chemical resistance after damage.

#### 2.5.3 Chemical resistance before damage and regrowth tolerance

Likewise, we ran an LMM with square root‐transformed regrowth rate as the response variable and log‐transformed initial foliar cardenolide concentrations, CO_2_ treatment, and milkweed species as fixed effects. An interaction between initial foliar cardenolide concentration and CO_2_ indicates a difference between atmospheres in the relationship between initial plant chemical resistance and regrowth.

#### 2.5.4 Regrowth tolerance and the mechanically induced resistance of regrowth tissues

Lastly, we ran an LMM with log‐transformed regrowth foliar cardenolide concentrations as the response variable and square root‐transformed regrowth rate, CO_2_ treatment, and milkweed species as fixed effects. A significant interaction between CO_2_ treatment and regrowth rate would signify a difference between the two atmospheres in any correlation between the two defense traits.

### 2.6 Elevated CO_2_, milkweed species, and plant growth and resistance profiles

While the trade‐off model framework described above provided some information on how growth rates and chemical resistance responded to our treatments, we also performed the following additional analyses to ask further questions about defense phenotypes. To determine the effects of our treatments on plant growth rate prior to damage and regrowth rate after damage, we used CO_2_ treatment, the probability of regrowth, and milkweed species as fixed effects and square root‐transformed growth rates (mg/day) as response variables. Not all milkweed individuals regrew following damage. We therefore used generalized linear mixed models with binomial error distributions and logit link functions to assess the effects of plant species and CO_2_ treatment on the proportion of milkweed plants that regrew following damage.

We then examined how CO_2_ treatment and species influenced the relationship between growth rate prior to damage and regrowth rate following damage, using an LMM with square root‐transformed regrowth rate as the response variable and square root‐transformed initial growth rate, CO_2_ treatment, and species as fixed effects.

Plant chemical defense encompasses not only the total concentration of defense compounds but also the diversity of chemical species produced. We therefore examined the relationships between cardenolide community diversity and growth rates. We calculated cardenolide diversity using the Shannon diversity index borrowed from the biodiversity literature: H = −sum (P_i_log [P_i_]) where P_i_ is the relative amount of a cardenolide peak compared to the total amount of cardenolides in an individual plant (Rasmann & Agrawal, 2011). Similar to above, we selected simplified models from two starting LMMs: (a) with constitutive foliar cardenolide concentrations as the dependent variable and square root‐transformed growth rate prior to clipping, CO_2_ treatment, and milkweed species as fixed effects; and (b) with mechanically induced foliar cardenolide concentrations as the dependent variable and square root‐transformed regrowth rate, CO_2_ treatment, and milkweed species as fixed effects.

To compare the effects of CO_2_ treatment, and milkweed species on the community of cardenolide compounds produced in the plants before and after damage, we used permutational multivariate analysis of variance (PerMANOVA; Anderson, 2001). The model included CO_2_ treatment, milkweed species, tissue type, and their interactions as fixed effects, and Bray–Curtis distance of percentage weight of each foliar cardenolide peak as dependent variables. To visualize these differences, we used nonmetric multidimensional scaling (NMDS) with 999 permutations per model run and a maximum of 500 runs per dimension (model stress = 0.200). PerMANOVA and NMDS scaling were performed using the VEGAN package in R (Oksanen & Friendly, 2017).

## 3 RESULTS

### 3.1 Only regrowth tolerance and induced resistance traded off among individuals following mechanical damage

#### 3.1.1 Plant growth rate and constitutive resistance

**Figure 1:**
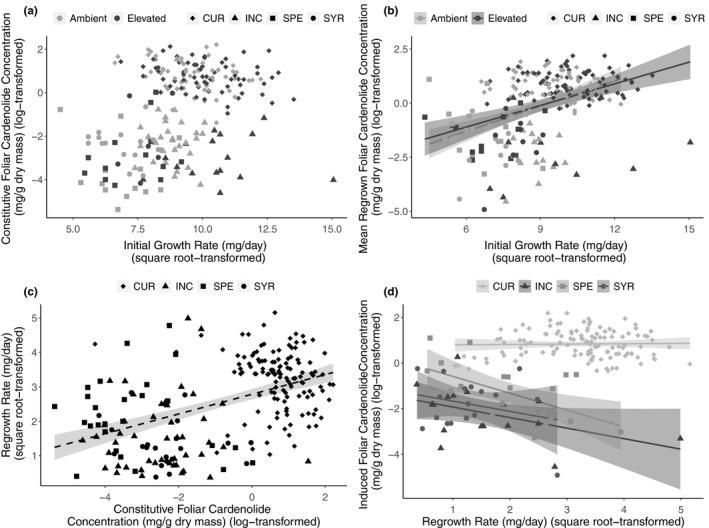
Support for only one of the four hypothetical trade‐offs relating the growth rate of milkweeds before and after damage to their constitutive and mechanically induced foliar cardenolide concentrations before and after damage. Milkweeds were grown under either elevated (760 ppm) or ambient (400ppm) atmospheric concentrations of CO_2_. (a) Nonsignificant effects of CO_2_ treatment and predamage growth rate on milkweed constitutive cardenolide concentrations (mg/g dry mass) before damage. (b) Positive effects of CO_2_ treatment and predamage growth rate on mechanically induced cardenolide concentrations in regrown leaves (mg/g dry mass) after damage. (c) Slight effects of milkweed constitutive cardenolide concentrations before damage on regrowth rate after damage contingent upon milkweed species. (d) Significant trade‐off between mechanically induced cardenolide concentrations (mg/g dry mass) in regrowth leaves and the regrowth rate (mg/day) of milkweeds. Regressions are represented with 95% confidence intervals and milkweed species codes are as follows: CUR = *A. curassavica* (diamond), INC = *A. incarnata* (square), SPE = *A. speciosa* (triangle), and SYR = *A. syriaca* (circle). In figures (a–c), light gray shapes represent plants grown under ambient CO_2_ and dark gray shapes are those grown under elevated CO_2_. In figure (d) shading corresponds to milkweed species

Milkweed growth rate prior to damage was unrelated to foliar constitutive cardenolide concentrations prior to damage (initial growth rate: F_1,195_ = 2.72, *p* = .100, Figure 1a; Table 2). Elevated CO_2_ had no effect on this nonsignificant relationship (CO_2_*initial growth rate: F_1,195_ = 0.46, *p* = .499).

**Table 2:** ANOVA tables of linear mixed effects models used to investigate the four putative trade‐offs proposed in this study

Trade‐off 1: constitutive resistance ~ species + CO_2_ + sqrt (growth rate) + CO_2_*sqrt (growth rate) + random = chamber						
	species	CO_2_	sqrt (growth rate)	species*CO_2_	atm*sqrt (growth rate)	Random Effect ± SD
F	F_3,207_ = 189.32	F_1,193_ = 0.29	F_1,195_ = 2.72	F_3,207_ = 3.84	F_1,195_ = 0.46	chamber
p	< 0.0001	0.59346	0.10044	0.01047	0.49931	0.06885 ± 0.2624

Trade‐off 2: induced resistance ~ sqrt (growth rate) + species + CO_2_ + CO_2_*sqrt (growth rate) + random = chamber						
	sqrt (growth rate)	species	CO_2_	species*sqrt (growth rate)	CO_2_*sqrt (growth rate)	Random Effect ± SD
F	F_1,214_ = 0.58	F_3,215_ = 8.59	F_1,213_ = 4.90	F_3,215_ = 1.73	F_1,215_ = 5.33	chamber
p	0.44782	< 0.0001	0.028	0.16224	0.02188	0.0003061 ± 0.0175

Trade‐off 3: sqrt (regrowth rate) ~ log (constitutive) + species + CO_2_ + species*CO_2_ + random = chamber					
	log (constitutive)	species	CO_2_	species* CO_2_	Random Effect ± SD
F	F_1,208_ = 3.66	F_3,208_ = 24.27	F_1,61_ = 0.09	F_3,207_ = 0.83	chamber
p	0.05716	< 0.0001	0.77115	0.47673	0.02114 ± 0.1454

Trade‐off 4: induced resistance ~ sqrt (regrowth rate) + species + CO_2_ + species*sqrt (regrowth rate) + random = chamber					
	sqrt (regrowth rate)	species	CO_2_	species*sqrt (regrowth rate)	Random Effect ± SD
F	F_1,216_ = 21.11	F_3,216_ = 14.37	F_1,45_ = 0.16	F_3,215_ = 7.18	chamber
p	<0.0001	<0.0001	0.6879328	0.00013	0.007618 ± 0.08728

Note

Model selection was performed using maximum likelihood. Tables were produced with the R package LmerTest, using type III sums of squares with Satterthwaite approximation for degrees of freedom, random effects estimates ± 1 standard deviation, and fixed effects parameter estimates ± 1 standard deviation.

#### 3.1.2 Plant growth rate before damage and mechanically induced resistance

Instead of a trade‐off between growth rate prior to damage and the mechanically induced chemical resistance of regrown tissues following damage, we found a positive relationship that weakened (became less steep) under elevated CO_2_ (CO_2_*initial growth rate: F_1,215_ = 5.33, *p* = .022, Figure 1b; Table 2).

#### 3.1.3 Constitutive resistance before damage and regrowth tolerance after damage

Similarly, we observed a weak positive relationship between constitutive chemical resistance and regrowth tolerance (constitutive resistance: F_1,208_ = 3.66, *p* = .057, Figure 1c; Table 2). Model selection eliminated models containing the influence of CO_2_ on this relationship.

#### 3.1.4 Regrowth tolerance and mechanically induced resistance of regrown tissues

In contrast to the first three potential trade‐offs, we observed a significant trade‐off between regrowth tolerance and the mechanically induced chemical resistance of regrown foliage (Regrowth rate*milkweed species: F_1,215_ = 7.18, *p* = .0001, Figure 1d; Table 2). The trade‐off was determined by two of the four milkweed species (*A. incarnata* and *A. speciosa*). As above, our selection process eliminated models containing the influence of CO_2_ on this relationship.

### 3.2 Elevated CO_2_ eliminated the positive relationship between initial growth rate and regrowth tolerance following damage

**Figure 2:**
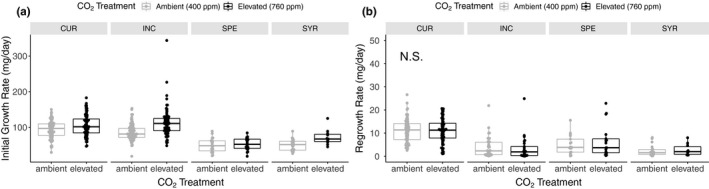
Elevated CO_2_ increased initial milkweed growth rate but had no lasting effects on regrowth rate following damage. The effects of CO_2_ treatment and milkweed species on (a) initial growth rate prior to damage (mg dry mass of above‐ground tissue/64 days) and (b) nonsignificant effects of elevated CO_2_ and milkweed species on regrowth rate following damage (mg dry mass of above‐ground tissue/21 days). In boxplots, dark lines represent the median, box boundaries represent first and third quartiles, and whiskers extend to the most extreme data point less than 1.5 times the interquartile range from the box. Milkweed species codes are the same as above. Data are grouped by species and CO_2_ treatment for ease of interpretation; however, the interaction term was not retained in our models of regrown plants

Across all milkweed species, elevated CO_2_ induced an average 24% increase in growth rate (CO_2_: F_1,151_ = 9.71, *p* = .002, Figure 2a) illustrating the classic effect of CO_2_ fertilization on plant growth (Kimball, 1983;Leadley, Niklaus, Stocker, & Körner, 1999). Initial growth rates of milkweed increased most strongly in *A. syriaca* (43%) followed by *A. incarnata* (31%), *A. curassavica* (12%), and *A. speciosa* (7%) (species*CO_2_: F_3,409_ = 3.24, *p* = .022, Figure 2a). Surprisingly, previous CO_2_ exposure had no effect on regrowth tolerance across milkweed species (CO_2_: F_1, 61_ = 0.09, *p* = .77, Figure 2b; Table 2) nor was there an interaction between species and CO_2_ treatment on milkweed regrowth tolerance (species*CO_2_: F_3, 207_ = 0.83, *p* = .477, Table 2). This result contradicted our original prediction that increased carbon availability and reduced water loss under elevated CO_2_ would favor faster rates of regrowth in damaged plants. Milkweed regrowth rate following damage was highest in *A. curassavica* (10.05 ± 0.45 mg/day) and lowest in *A. syriaca* (2.12 ± 0.34 mg/day) (species: F_3,208_ = 24.27, *p* < .0001, Figure 2b; Table 2).

**Figure 3:**
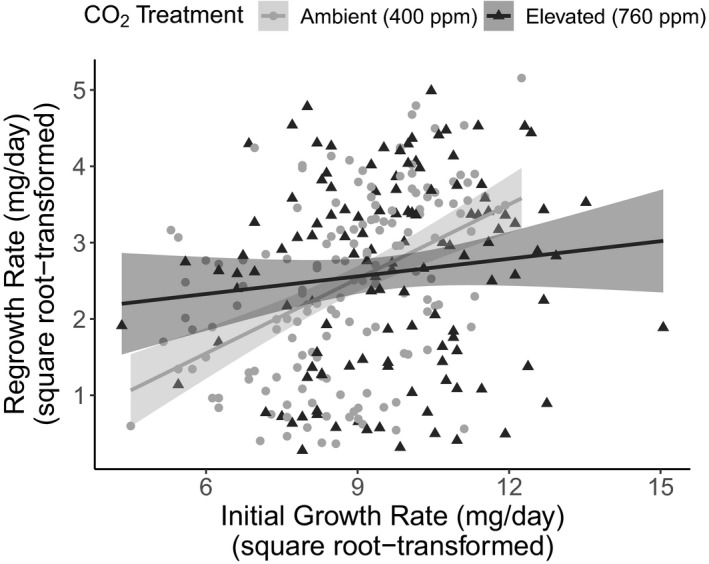
Elevated CO_2_ uncoupled the positive relationship between initial plant growth rate and regrowth rate following damage. Light gray circles and lines represent plants grown under ambient CO_2_ and dark gray triangles and lines are those grown under elevated CO_2_. Regressions are represented with 95% confidence intervals

**Figure 4:**
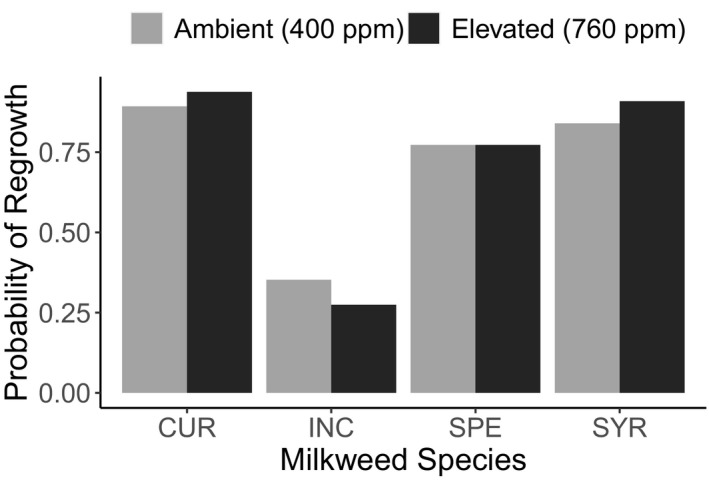
Variation among milkweed species in the probability of regrowth after mechanical damage. Light gray bars represent plants grown under ambient CO_2_ and dark gray bars are those grown under elevated CO_2_. Data are grouped by species and CO_2_ treatment for ease of interpretation; however, the interaction term was not significant in the models. Milkweed species codes are the same as above

Intriguingly, elevated CO_2_ weakened the positive relationship between initial plant growth rate and regrowth rate following damage (Regrowth rate* CO_2_: F_1, 263_ = 5.99, *p* = .015, Figure 3; Table 3). In other words, future atmospheric concentrations of CO_2_ uncoupled the relationship between regrowth tolerance following damage and initial growth rate before damage. Following mechanical damage, only 278 of the 442 plants (63%) regrew aboveground tissue. Despite previous carbon supplementation, elevated CO_2_ did not affect the probability of regrowth (χ^2^ = 0.16, *p* = .6875, Figure 4) nor was there an interaction between milkweed species and CO_2_ treatment on regrowth probability (χ^2^ = 1.47, *p* = .689, Figure 4).

**Table 3:** ANOVA table of a linear mixed effects model describing the effects of elevated CO_2_ on the relationship between initial plant growth rate and regrowth rate following damage

Model: sqrt (regrowth rate) ~ sqrt (growth rate) + CO_2_ + species + species* sqrt (growth rate) + CO_2_* sqrt (growth rate) + random = chamber							
	sqrt (growth rate)	species	CO_2_	species*sqrt (growth rate)	species* CO_2_	CO_2_*sqrt (growth rate)	Random Effect ± SD
F	F_1,261_ = 0.01	**F_3,257_ = 2.88**	**F_1,260_ = 5.95**	F_3,257_ = 2.14	F3_,260_ = 1.17	**F_1,263_ = 5.99**	chamber
p	0.90362	**0.03633**	**0.01543**	0.09542	0.32088	**0.01505**	0.07448 ± 0.2729

Note

As above, model selection was performed using maximum likelihood. Tables were produced with the R package LmerTest, using type III sums of squares with Satterthwaite approximation for degrees of freedom, random effects estimates ± 1 standard deviation, and fixed effects parameter estimates ± 1 standard deviation.

### 3.3 Elevated CO_2_ altered the magnitude and diversity of chemical resistance

**Figure 5:**
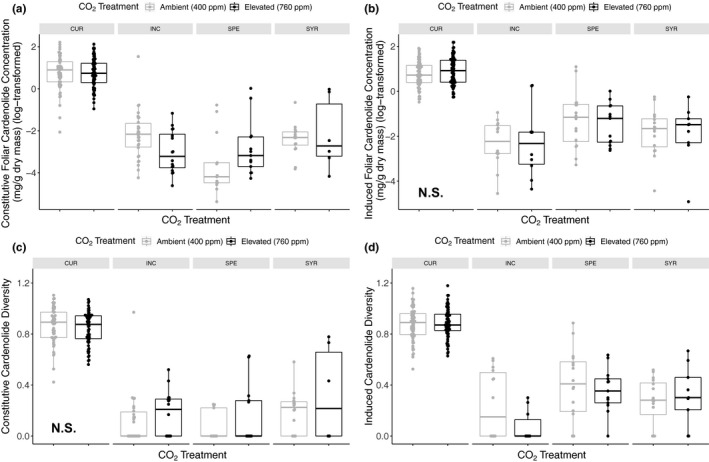
Elevated CO_2_ altered the total concentration of milkweed constitutive defense and the diversity of mechanically induced defense following damage. (a) The effects of elevated atmospheric concentrations of CO_2_ on constitutive cardenolide concentrations of milkweed (mg/g dry mass), (b) the mechanically induced cardenolide concentrations of milkweeds (mg/g dry mass), (c) the diversity of cardenolides produced constitutively, and (d) the diversity of cardenolides produced in the mechanically induced resistance response following damage. Data are grouped by species and CO_2_ treatment for ease of interpretation; however, the interaction term was not retained in our models for B and C. Dark gray points represent plants grown under elevated CO_2_ and light gray points and lines are those grown under ambient CO_2_. Milkweed species codes are the same as above

Elevated CO_2_ reduced constitutive resistance in *A. incarnata* by 37%, in *A. syriaca* by 10%, slightly in *A. curassavica* by 5%, and increased constitutive resistance in *A. speciosa* by 22% (species*CO_2_: F_3,207_ = 3.84, *p* = .010, Figure 5a; Table 2). Milkweed species was by far the most important determinant of constitutive cardenolide concentration (species: F_3,207_ = 189.32, *p* < .0001, Figure 5a; Table 2). In those plants that did regrow following damage, mechanically induced resistance varied substantially by milkweed species (species: F_3,215_ = 8.59, *p* < .0001, Figure 5b; Table 2). *A. curassavica* again produced the highest concentrations of foliar cardenolides, followed by *A. speciosa, A. syriaca,* and *A. incarnata.* Across all four species, mechanically induced resistance increased by 28% in those plants previously exposed to elevated CO_2_ (CO_2_: F_1,213_ = 4.90 *p* = .028, Figure 5b; Table 2).

The diversity of cardenolides produced constitutively among milkweed species increased by 24% under elevated CO_2_ (CO_2_: F_1,68_ = 4.08, *p* = .047, Figure 5c; Table 4). Despite a species‐specific effect of elevated CO_2_ on the total concentration of constitutive resistance, there was no such effect on the diversity of cardenolides produced constitutively (species*CO_2_: F_3,206_ = 2.04, *p* = .109, Figure 5c; Table 4). Conversely, the diversity of cardenolides produced in the mechanically induced resistance profiles of both *A. incarnata,* and *A. speciosa* declined by 70% and 11% after previous exposure to elevated CO_2_ (species*CO_2_: F_3,20_ = 2.67, *p* = .048, Figure 5d; Table 4).

**Table 4:** ANOVA tables of linear mixed effects models describing the relationships between the diversity of constitutive and induced cardenolides and growth rates dependent on milkweed species and elevated CO_2_

Model: constitutive diversity ~ sqrt (growth rate) + species + CO_2_ + species*CO_2_ + random = chamber					
	sqrt (growth rate)	species	CO_2_	species* CO_2_	Random Effect ± SD
F	F_1,201_ = 0.76	F_3,207_ = 260.56	F_1,68_ = 4.077	F_3,206_ = 2.04	chamber
p	0.38452	<0.0001	0.04741	0.10937	0.003 ± 0.054

Model: induced diversity ~ sqrt (regrowth rate) + species + CO_2_ + sqrt (regrowth rate)*species + species*CO_2_ + random = chamber						
	sqrt (regrowth rate)	species	CO_2_	sqrt (regrowth rate)*species	species* CO_2_	Random Effect ± SD
F	F_1,211_ = 1.94	F_3,205_ = 29.67	F_1,72_ = 1.95	F_3,206_ = 4.62	F_3,203_ = 2.67	chamber
p	0.1646	<0.0001	0.16661	0.003752	0.04841	0.003 ± 0.057

Note:

As above, model selection was performed using maximum likelihood. Tables were produced with the R package LmerTest, using type III sums of squares with Satterthwaite approximation for degrees of freedom, random effects estimates ± 1 standard deviation, and fixed effects parameter estimates ± 1 standard deviation.

**Figure 6:**
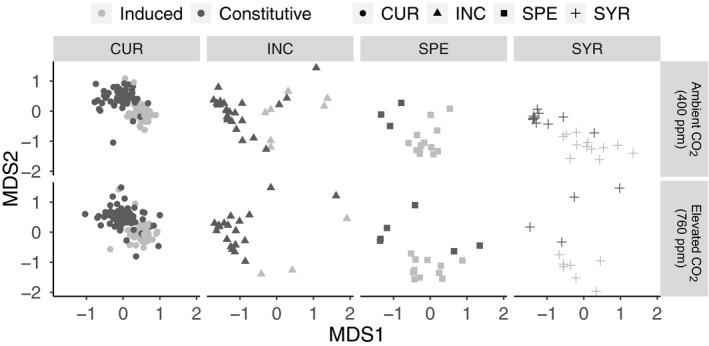
Previous exposure to elevated CO_2_ caused slight changes in both the constitutive and mechanically induced cardenolide communities of milkweed. Dark gray points represent the constitutive cardenolide communities produced by plants before damage, and light gray points are the cardenolide communities detected in the mechanically induced response of milkweed following damage. Those plants grown under ambient CO_2_ are in the upper panel and those grown under elevated CO_2_ are in the lower panel. Milkweed species codes are the same as above

When comparing the composition of cardenolide communities among individuals before and after damage, the difference between constitutive and mechanically induced foliar tissue was the strongest driver of community dissimilarity as determined by PerMANOVA (resistance type: F_1, 410_ = 55.38, *p* = .001, R^2^ = 0.15, Figure 6; Table 5). There were slight differences between these two resistance profiles among milkweed species driven by elevated CO_2_ (resistance type*species*CO_2_: F_2, 410_ = 2.39, *p* = .001, R^2^ = 0.013, Figure 6; Table 5), and these slight differences likely represent the changes in cardenolide diversity detected above.

**Table 5:** PerMANOVA describing the effects of elevated CO_2_ on the composition of constitutive and induced cardenolide communities

PerMANOVA			
	F	*R^2^*	*P*
species	F_3,410_ = 12.12	0.10	.001
CO_2_	F_1,410_ = 1.06	0.003	.404
resistance type	F_1,410_ = *55.38*	0.15	.001
resistance type*species	F_3,410_ = 4.74	0.04	.001
resistance type*CO_2_	F_1,410_ = 1.04	0.003	.391
species*CO_2_	F_2,410_ = 2.26	0.011	.003
resistance type*species*CO_2_	F_2,410_ = 2.39	0.013	.001

## 4 DISCUSSION

Our study reveals the limitations of a trade‐off framework at the physiological level when considering how complex defense phenotypes respond to environmental change. Of the four hypothesized trade‐offs among aspects of plant growth and resistance framing the study, we found support for only one between regrowth tolerance and mechanically induced chemical resistance (foliar cardenolide concentration following mechanical damage). The strength of this trade‐off was unaffected by previous exposure to elevated CO_2_ but varied substantially among milkweed species, presumably reflecting species‐specific allocation patterns to defense following damage. In contrast to expected trade‐offs, we found positive relationships among some growth and resistance traits. However, the positive relationship between growth rate prior to damage and mechanically induced chemical resistance was weaker under previous exposure to elevated CO_2_. Our data add to a growing body of work that demonstrates the complex nature of plant growth and resistance relationships and highlights the need to test allocation strategies of plants in the context of rapidly changing environmental resources on ecological time scales as well as across evolutionary contexts.

Multiple mechanisms may govern the direction and magnitude of growth and resistance relationships in plants. These mechanisms include nutrient limitation, allocation costs, genetic linkage of defense traits, and ecological costs (Boege, Dirzo, Siemens, & Brown, 2007;Fine, Miller, & Mesones, 2006;Simms & Rausher, 1987;Strauss, Siemens, Decher, & Mitchell‐Olds, 1999;Tao et al., 2016;Tucker & Avila‐Sakar, 2010;Wise & Abrahamson, 2007;Züst & Agrawal, 2017). Among plants that regrew following damage, we found evidence of a trade‐off between mechanically induced cardenolide concentrations and regrowth tolerance in three of four milkweed species (Figure 1d). This finding supports previous studies that have reported negative relationships between milkweed growth and cardenolide production (Hochwender et al., 2000;Tao et al., 2016;Züst, Rasmann, & Agrawal, 2015). However, ours is the first study within the milkweed system to show interspecific differences in regrowth tolerance and mechanically induced resistance relationships following damage. Interestingly, previous exposure to elevated CO_2_ had no effect on the strength of this trade‐off, indicating that the legacy of carbon supplementation in isolation may not be a critical driver of plant induced defense syndromes. Only the tropical *A. curassavica*, native to central America, failed to display a trade‐off between mechanically induced resistance and regrowth tolerance. Higher herbivore pressure at southern latitudes may select for higher levels of both defense traits in this species as compared to the other three perennials native to N. America (Rasmann & Agrawal, 2011). The positive relationship between innate plant growth and mechanically induced resistance could also reflect selection for vigorous plants capable of mounting a strong response to herbivory (Hahn et al., 2019; Figure 1c). Interestingly, with faster predamage growth rates under elevated CO_2_, plants produced lower levels of induced resistance likely as a result of suppressed phytohormonal signaling pathways (Guo et al., 2012;Ode et al., 2014).

Despite finding no influence of elevated CO_2_ on three of the four relationships between growth and resistance in our study, elevated CO_2_ altered aspects of both milkweed growth and resistance independently. Notably, elevated CO_2_ uncoupled the positive relationship between initial plant growth rate and regrowth tolerance following damage (Figure 3). Often plants with high innate growth rates can regrow faster following damage (Rosenthal & Kotanen, 1994). However, in our study, those plants that were fast growing under elevated CO_2_ did not maintain a proportionately high level of regrowth under ambient CO_2_ following damage. Because the regrowth period took place in a greenhouse under ambient CO_2_ with homogenous soil nutrients and water availability, these data potentially indicate the legacy of elevated CO_2_ in altering phytohormonal signaling pathways responsible for regrowth tolerance (Guo et al., 2012). The constitutive resistance of both *A. incarnata* and *A. syriaca* declined under elevated CO_2_ and increased in *A. speciosa*. Despite these effects of elevated CO_2_ on constitutive defense, no legacy of this treatment was detected in the mechanically induced resistance response of the milkweed species. Such conserved induction responses despite previous exposure to elevated CO_2_ suggests that changes in chemical resistance due to elevated CO_2_ detected by this and other studies (Ode et al., 2014;Zavala, Gog, & Giacometti, 2017;Zavala et al., 2013) rely on continuous carbon supplementation and simultaneous manipulation of phytohormonal signaling pathways rather than previous allocation decisions made by the plant before damage.

Monarch caterpillars are iconic milkweed herbivores undergoing significant declines, due, in part, to changing environmental conditions in both overwintering and summer breeding grounds (Stephen Malcolm, 2017;Stenoien et al., 2016). Roadside milkweed patches are important habitat for monarchs and regularly experience mowing events (Kasten, Stenoien, Caldwell, & Oberhauser, 2016;Mueller & Baum, 2014). Appropriately timed mowing treatments can increase monarch fecundity within milkweed patches by increasing the availability of high‐quality foliage and releasing monarchs from the presence of enemies (Borkin, 1982;Fischer, Williams, Brower, & Palmiotto, 2015;Haan & Landis, 2019;Knight, Norris, Derbyshire, & Flockhart, 2019). Our study reveals that elevated CO_2_ changes the composition and reduces the diversity of cardenolides produced after simulated mowing in both *A. incarnata,* and *A. speciosa*, two milkweed species commonly found in the N. American summer breeding grounds (Woodson, 1954). Critically, the composition of cardenolide communities produced by milkweed can alter monarch interactions with natural enemies, such as a prevalent protozoan pathogen (Decker, Roode, & Hunter, 2018;Decker, Soule, Roode, & Hunter, 2019;Sternberg et al., 2012). Given the conservation importance of roadside milkweed patches that are regularly mowed throughout N. America, changes in regrowth tissue chemical quality could have implications for monarch populations. Yet, attempts to predict how migratory monarchs that depend on roadside milkweed corridors will perform under global environmental change remain challenging (Zipkin, Ries, Reeves, Regetz, & Oberhauser, 2012).

Our study, though comprehensive in its investigation of growth and chemical resistance before and after damage, does not incorporate the entire suite of defenses expressed by milkweeds. Additional direct and indirect defenses include trichomes, latex, leaf toughness, and volatile emissions that attract natural enemies (Agrawal & Fishbein, 2006;Agrawal & Konno, 2009;Hochwender et al., 2000;Meier & Hunter, 2019;Zalucki et al., 2001). This suite of defense strategies may also generate resource‐based trade‐offs and alter plant‐herbivore interactions (Züst & Agrawal, 2017;Züst et al., 2015). Thus, further studies exploring the fitness costs of regrowth tolerance and multiple defenses under future environmental conditions, and the responses of herbivore populations to these changes, are greatly needed.

On an evolutionary timescale, the influence of resource clines has illustrated the existence of trade‐offs between growth and resistance, lending broad support to the RAH (Coley & Chapin, 1985;Endara & Coley, 2011;Strauss & Agrawal, 1999). Currently, no well‐established theory makes predictions about how trade‐offs among defense traits will respond to rapid environmental change within one generation. In our study, the identity of the milkweed species determined our ability to detect a trade‐off between regrowth tolerance and resistance following mechanical damage, and previous exposure to elevated CO_2_ weakened a positive relationship between innate growth rate and constitutive defense. Given the rapid rate of environmental change predicted globally (Stocker et al., 2013), studies measuring the rate of plant resistance and growth evolution as well as which environmental change drivers are crucial determinants of plant fitness will be vital to predicting plant‐insect interactions. This knowledge can be used to inform policy decisions which reduce the use of pesticides (Strauss & Murch, 2004) and improve weed control programs (Williams, Walsh, & Boydston, 2004).

## CONFLICT OF INTEREST

Authors have no sources of conflict of interest.

## AUTHOR CONTRIBUTION

**Leslie E Decker:** Conceptualization (equal); Data curation (lead); Formal analysis (lead); Funding acquisition (supporting); Investigation (lead); Methodology (equal); Project administration (lead); Resources (equal); Visualization (lead); Writing‐original draft (lead); Writing‐review & editing (lead). **Mark D. Hunter:** Conceptualization (equal); Data curation (supporting); Formal analysis (supporting); Funding acquisition (lead); Investigation (supporting); Methodology (equal); Project administration (supporting); Resources (lead); Supervision (equal); Visualization (supporting); Writing‐original draft (supporting); Writing‐review & editing (supporting). 

## Data Availability

Data are available in the Dryad Digital Repository: https://doi.org/10.5061/dryad.v6wwpzgs3
